# The use of wheatgrass (Thinopyrum intermedium) in breeding

**DOI:** 10.18699/VJGB-22-51

**Published:** 2022-08

**Authors:** I.V. Pototskaya, V.P. Shamanin, A.N. Aydarov, A.I. Morgounov

**Affiliations:** Omsk State Agrarian University named after P.A. Stolypin, Omsk, Russia; Omsk State Agrarian University named after P.A. Stolypin, Omsk, Russia; Omsk State Agrarian University named after P.A. Stolypin, Omsk, Russia; Food and Agriculture Organization, Riyadh, Saudi Arabia

**Keywords:** perennial crop, wheat, domestication, selection, genes, ecology, многолетняя культура, пшеница, доместикация, отбор, гены, экология

## Abstract

Wheatgrass (Th. intermedium) has been traditionally used in wheat breeding for obtaining wheat-wheatgrass hybrids and varieties with introgressions of new genes for economically valuable traits. However, in the 1980s in the United States wheatgrass was selected from among perennial plant species as having promise for domestication and the development of dual-purpose varieties for grain (as an alternative to perennial wheat) and hay. The result of this work was the creation of the wheatgrass varieties Kernza (The Land Institute, Kansas) and MN-Clearwater (University of Minnesota, Minnesota). In Omsk State Agrarian University, the variety Sova was developed by mass selection of the most winter-hardy biotypes with their subsequent combination from the population of wheatgrass obtained from The Land Institute. The average grain yield of the variety Sova is 9.2 dt/ha, green mass is 210.0 dt/ ha, and hay is 71.0 dt/ha. Wheatgrass is a crop with a large production potential, benef icial environmental properties, and valuable grain for functional food. Many publications show the advantages of growing the Kernza variety compared to annual crops in reducing groundwater nitrate contamination, increasing soil carbon sequestration, and reducing energy and economic costs. However, breeding programs for domestication of perennial crops are very limited in Russia. This paper presents an overview of main tasks faced by breeders, aimed at enhancing the yield and cultivating wheatgrass eff iciency as a perennial grain and fodder crop. To address them, both traditional and modern biotechnological and molecular cytogenetic approaches are used. The most important task is to transfer target genes of Th. intermedium to modern wheat varieties and decrease the level of chromatin carrying undesirable genes of the wild relative. The f irst consensus map of wheatgrass containing 10,029 markers was obtained, which is important for searching for genes and their introgressions to the wheat genome. The results of research on the nutritional and technological properties of wheatgrass grain for the development of food products as well as the differences in the quality of wheatgrass grain and wheat grain are presented.

## Abstract (ru)

Пырей средний (Th. intermedium) традиционно применялся в селекции пшеницы для получения пшенично-пырейных гибридов и сортов с интрогрессиями новых генов хозяйственно ценных признаков. Однако в 1980-х гг. в США из множества многолетних видов растений пырей был выбран для доместикации с целью создания сортов двойного направления использования – на зерно (альтернатива многолетней пшенице) и сено. В результате были выведены сорта пырея Kеrnza (The Land Institute, Канзас) и MN-Clearwater (Миннесотский университет, Миннесота). В Омском ГАУ из популяции Th. intermedium, полученной из The Land Institute, массовым отбором наиболее зимостойких биотипов с последующим их объединением создан сорт Сова. Средняя урожайность зерна сорта Сова составляет 9.2 ц/га, зеленой массы – 210.0 ц/га, сена – 71.0 ц/ га. Пырей средний – культура с большим производственным потенциалом, полезными экологическими свойствами и ценным зерном для функционального питания. Во многих публикациях показаны преимущества возделывания сорта Kеrnza по сравнению с однолетними культурами: сокращение стока нитратов в грунтовые воды, увеличение секвестрации почвенного углерода, снижение энергетических и экономических затрат. Однако в России селекционные программы, направленные на доместикацию многолетних культур, весьма ограниченны. В настоящем обзоре рассматриваются основные задачи, стоящие перед селекцией и направленные на повышение урожайности зерна и эффективности возделывания пырея среднего в качестве много- летней зерновой и кормовой культуры. Для их решения используются как традиционные, так и современные биотехнологические и молекулярно-цитогенетические подходы. Важнейшей задачей считается передача це- левых генов Th. intermedium в современные сорта пшеницы и сокращение дозы хроматина, несущего гены нежелательных признаков дикорастущего сородича. Получена первая консенсусная генетическая карта пы- рея среднего, содержащая 10 029 маркеров и представляющая интерес для поиска ценных генов и их интро- дукции в геном пшеницы. Представлены результаты исследований по оценке питательных и технологических свойств зерна пырея и полученных из него продуктов питания в сравнении с пшеницей.

## Introduction

Climate change is an urgent problem affecting food security, since in the arid agricultural landscapes of Africa, Asia, and the South America cereals yield is sharply decreasing, in particular that of maize, wheat, and sugar beetroot (IPCC…, 2019).

The traditional agricultural system based on the cultivation of annual crops implies the usage of pesticides and moldboard plow tillage, which significantly reduces its fertility, leads to erosion of arable land, leaching of nutrients, and carbon emissions (Stavridou et al., 2016; Vico, Brunsell, 2018). About 70 % of total greenhouse gas emissions (CO2, CH4, N2O, etc.) account for the application and production of nitrogen fertilizers, 10–15 % – for agrotechnical methods of tillage, the rest – for the usage of pesticides and growth regulators (Berry et al., 2010).

Annual crops occupy more than three quarters of the crops area in the world according to the latest data, so an important element of regenerative agriculture is creating a rational structure of cultivated areas and increasing the biodiversity of cultivated crops (de Oliveira et al., 2019). In the coming decades, the expansion of cultivated areas under perennial crops, in addition to annual crops, will create opportunities for transitioning agriculture to a more sustainable development trajectory, reduce production costs, and improve the agrocenoses state (Amaducci et al., 2016).

Perennial crops have a longer growing period, due to which the soil is covered with vegetation longer, provide carbon accumulation in the soil, and reduce greenhouse gas emissions (Chimento, Amaducci, 2015; Schipanski et al., 2016). They have increased resistance to many negative biotic and abiotic environmental factors, form a powerful root system that improves plant water consumption and reduce nutrient losses in the soil (Zeri et al., 2013; Abraha et al., 2016). The wheatgrass Thinopyrum intermedium (Host) Barkworth & D.R. Dewey and oilseed culture Silphium integrifolium Michx. are examples of successful domestication of perennial crops. Wide hybridization of annual crops with perennial wild relatives is carried out in many scientific institutions and universities around the world to create such perennial crops as wheat, sorghum, rice, barley (Crews et al., 2018).

## Biological and genetic properties of Intermediate wheatgrass (IWG)

Allohexaploid species Th. intermedium (2n = 6 x = 42) (= syn. Agropyron glaucum (Desf. ex DC.) Roem. & Schult. = Elytrigia intermedia (Host) Nevski) is a perennial wild species characterized by a wide variety of morphological properties and high adaptability to biotic and abiotic stresses (Razmakhnin, 2008). This species is included in the tertiary gene pool, differs from all other species of the genus Thinopyrum A. Löve by high crossbreeding with bread wheat (average grain percentage of hybrids is 24) (Gill et al., 2006; Cui et al., 2018). However, the transfer of valuable genes from IWG to bread wheat is difficult, which is explained by limited recombination between chromosomes of these species in distant hybrids. Four main methods are used for targeted introgressions from the homeologous chromosomes of wild wheat relatives to the wheat genome: spontaneous translocations, radiation exposure, tissue culture, and induced homeologous recombination. The last method is used provided that the target gene is removed from the near centromeric regions where recombination is absent or difficult (Zhang P. et al., 2017).

The genomic composition of Th. intermedium (JJSSt) has been studied for decades. The results of genomic in situ hybridization (GISH) with usage of labeled DNA of different diploid species as probes showed that the J-genome is related to the genome of diploid species Th. bessarabicum and Th. elongatum, and the JS-genome – modified form of the genome Th. elongatum/Th. bessarabicum. The St-genome is the main genome of perennial grasses; it shows great similarity with the genome of genus Pseudoroegneria, which is the most probable maternal parent of Th. intermedium (Chen et al., 1998; Chen, 2005; Mahelka et al., 2011; Kroupin et al., 2019).

In the 1930s, scientists had great expectations for wide hybridization, when N.V. Tsitsin in the Soviet Union, as well as other scientists in the USA and Canada, began to develop perennial wheat forms by crossing bread wheat with IWG (Suneson et al., 1963; Tsitsin, 1978). In the Main Botanical Garden named after N.V. Tsitsin of the RAS (Moscow), under the leadership of academician N.V. Tsitsin was formed a unique collection, which included the octoploid forms of wheat-wheatgrass hybrids (WWGHs) obtained using different species of wheatgrass, as well as varieties Istra 1, Zernokormovaya 169, Ostankino, Otrastayutshaya 38 (Upelniek et al., 2012). For the first time, the winter bread wheat varieties characterized by medium level of winter hardiness were created on the basis of wheat-wheatgrass hybrids WWGH 599 and WWGH 186. In the 1970s, the variety Zarya was developed in the Federal Research Center “Nemchinovka”, which was cultivated on an area larger than 500 thousand hectares (Sandukhadze et al., 2021). Modern varieties and lines Multi 6R, Lebedushka, Belyanka of Samara ARI have a substituted chromosome 6D(6Agi); varieties Tulaykovskaya 5, 10, 100 of Saratov ARI have a substituted chromosome 6D(6Agi2) with highly effective resistance genes to brown, stem, yellow rust, and powdery mildew belonging to Th. intermedium (Sibikeev et al., 2005; Salina et al., 2015). In Western Siberia, some perspective WWGHs based on Th. intermedium and Ag. еlongatum were developed. They are recommended for inclusion in hybridization with varieties of winter and spring bread wheat in order to increase winter hardiness, resistance to rust diseases, and grain quality (Plotnikova et al., 2011; Razmakhnin et al., 2012). In China, since the early 1950s, systematic work has been carried out to increase wheat resistance to different abiotic and biotic environmental factors using Th. intermedium. The WWGHs with characteristics such as high winter hardiness, disease resistance, improved feed properties, and rapid post-harvest regrowth were involved in the breeding of perennial fodder wheat (Cui et al., 2018).

Biotechnological and molecular cytogenetic approaches to transfer the target gene to modern wheat varieties and reduce the unwanted alien chromatin of wild wheat relative are used (Kroupin et al., 2019). The genes of resistance to leaf, stem, yellow rust, powdery mildew (Lr38, Sr44, Yr50, Pm40, and Pm43), barley yellow dwarf virus (Bdv2, Bdv3), and wheat striped mosaic (Wsm1) were transferred to the wheat genome from IWG (Martynov et al., 2016; Ryan et al., 2018; Sibikeev et al., 2018).

Molecular markers for the analysis of the Th. intermedium genome, which makes it possible to purposefully transfer wheatgrass genes into the wheat genome, were developed (Kroupin et al., 2011; Li et al., 2016; Sibikeev et al., 2017). In particular, molecular markers have been developed to identify wheatgrass genes in the wheat genome: CAPS-marker for the Vp-1 gene is used in breeding to increase resistance to pre-harvest sprouting (Divashuk et al., 2011; Kocheshkova et al., 2017); CAPS-marker P22F/PRa/PvuII for the DREB1 gene, for the wheat drought tolerance breeding (Pochtovyi et al., 2013); molecular and cytogenetic markers specific to wheatgrass chromosome 1St#2, for breeding to increase the protein and gluten content in wheat grain (Li et al., 2013, 2016); WXTH-marker for the Wx gene, for changing starch composition and technological properties (Klimushina et al., 2020); PLUG, SCAR and Thi-GBS-markers, for identifying the chromosomes of the J-, JS-, and St-genomes of wheatgrass (Hu et al., 2012; Tang et al., 2020; Qiao et al., 2021).

Along with molecular markers, cytogenetic markers are effectively used to identify chromosomes and their segments belonging to Th. intermedium, which are associated with agronomic traits (Yu et al., 2019; Nikitina et al., 2020). The oligosondes (GAA)10, pSt122, pSc119.2-1, Oligo-B11, Oligo-pThp3.93, pAs1-1, pAs1-3, AFA-4 of the fluorescent (FISH) and genomic (GISH) hybridization are used to visualize Th. intermedium chromosomes in WWGHs and introgressive lines (Li et al., 2016; Xi et al., 2019; Wang et al., 2021). Three cytogenetically markers of tandem repeats, which were specific to Th. intermedium chromatin on different chromosomes of introgressive lines tolerant to phosphorus deficiency were developed (Zhang X. et al., 2021). The presence of reliable markers for wheatgrass chromosomes expands experimental possibilities for using this cereal in wheat breeding.

In 2016, the first consensus genetic map of IWG was obtained. It consists of 10,029 markers, each of 21 linkage groups contains between 237 and 683 markers with an average distance of 0.5 cM between each pair of markers (Kantarski et al., 2017). This map is of interest for identification of genes that control economically important agronomic traits and their introduction into the wheat genome. A total of 111 QTLs were detected for 17 variable traits in the M26 × M35 family including several large-effect QTLs responsible for seed retention, plant height, seed weight, seed threshing, and other economically important agronomic traits. By the method of association-mapping, 33 QTLs that control the grain size and weight were detected. When performing the selection of forms for seed weight, it was observed that the frequency of favorable QTL alleles in the IWG population was increased to >46 % (Larson et al., 2019).

## Breeding programs for the wheatgrass domestication

Domestication of a new species is a risky and unpredictable process, because during selection for target traits, one cannot be sure how other traits, desirable or undesirable for breeding, will change. In the 1980s, at the Rodale Research Center (Kutztown, USA), IWG was selected for domestication and seed production from over 100 perennial species. Among perennial crops, this cereal has relatively large seeds, moderate spike fragility, and good threshability, along with greater biomass and excellent quality of fodder (Wagoner, 1990; Becker et al., 1992). Two selection cycles according to agronomic characteristics and seed size were carried out. The perspective genets (clones) of wheatgrass were identified and transferred for further study to the Land Institute (Salina, Kansas, USA) (DeHaan et al., 2005; Cox et al., 2010).

At the Land Institute, the selection cycles began with the development of indices based on the characteristics: seed weight per plant, seed weight per spike, percent of the bare seed, thousand kernel weight, and disease damage. A population for over-pollination was formed corresponding to the indices in each selection cycle from 50–70 genets with the most favorable combination of traits. After two selection cycles, the grain yield per unit area increased by 77 %, and the seed weight, by 23 % (DeHaan et al., 2018). At the Land Institute and the University of Minnesota (Minnesota, USA), the results of genome sequencing (Thinopyrummedium v2.1 DOE-JGI, https://phytozome-next.jgi.doe.gov/info/Tintermedium_v2_1) were actively used for the domestication of Th. intermedium in order to replace time-consuming selection by phenotype by GWAS and bioinformatics methods (Bajgain et al., 2019; Crain et al., 2020, 2021).

As a result of many years of work at the Land Institute, the wheatgrass variety Kernza was developed (named after the residents of Kansas), used both for seed production, green mass, and hay (haylage). During the second year of the cultivating of the variety, there was an 86 % nitrate reduction in groundwater, and a 13 % increase in soil carbon sequestration compared to annual crops (Glover et al., 2010; Culman et al., 2013; DeHaan, Van Tassel, 2014; Pugliese et al., 2019). Kernza is practically not affected by diseases and pests, the crop requires fewer agrotechnical operations, such as nitrogen fertilizers, tillage, pre-sowing seed treatment, and fungicide protection, thereby reducing energy and economic costs (DeHaan et al., 2005; Pugliese et al., 2019).

During the cultivation period of Kernza in Kansas in 2012–2016, the nitrogen fertilization has changed over time, beginning with ∼110 kg per ha in 2012 and gradually decreasing to ∼80 kg per ha in 2016. For this period, carbon emissions were reduced from 513 to 121 g C·m−2. Over the whole study period, the total carbon fixed was ∼50 % higher than the carbon lost via respiration. Based on the cumulative net ecosystem exchange data (NEE), it was found that the perennial wheatgrass represented a substantial carbon sink 590.4 g C·m−2 per year (de Oliveira et al., 2018).

A five-year cultivation of the wheatgrass variety Kernza had positive effect on the soil structure and yield of the following crops in the crop rotation: it increased the microbiological activity and soil microbiota diversity compared to the soil microbiota under maize harvested for silage (Jungers et al., 2019). In comparison with annual crops such as maize and wheat, the variety Кеrnza also had a higher ability of maintaining the water-use efficiency (WUE) and evapotranspiration (ET) – about 97 % throughout the whole growing season. This was achieved thanks to a strong root system and water uptake from deeper soil layers, which is an important mechanism of adaptation to water deficit conditions (Suyker, Verma, 2009; Abraha et al., 2015; Sutherlin et al., 2019).

In 2011, a joint breeding program for improvement of Kernza was launched between the Land Institute and the University of Minnesota, which contributed to the commercial interest emergence for this perennial cereal. It was developed as a synthetic population at the University of Minnesota, prioritising grain-type direction, MN-Clearwater (experimental designation MN 1504), which can be cultivated for biomass and forage. Among 2,560 IWG genets received from the Land Institute, seven parents were selected according to the following set of characteristics: days to heading, plant height, spike weight, percentage free grain threshing, seed weight, and biomass weight to create a synthetic population of MN-Clearwater. In variety trials across Minnesota, MNClearwater produced 696 kg · ha−1, the thousand kernel weight was 6 g. This is a short-stemmed variety (113 cm), which had a good threshability (63 %), and low stem fragility with minimal lodging during research years (Bajgain et al., 2020). Programs for domestication and improvement of such IWG traits as seed size, threshability, reduction of spike fragility, and plant height for increasing resistance to lodging and diseases are also implemented at the University of Manitoba (Canada), at the University of Utah (USA), and at the University of Agricultural Sciences (Uppsala, Sweden) (Cattani, Asselin, 2016).

The introduction of optimal doses of fertilizers and appropriate agricultural technology increase the wheatgrass yield. Thus, in the autumn sowing of IWG population of grain-type (TLI-C2), grain yield was highest during the first year in response to nitrogen fertilization – 961 kg · ha−1 and gradually decreased in subsequent production years (Jungers et al., 2017). The experience of American farmers shows that IWG can be cultivated without replanting for 4–6 years, making a net profit by reducing production costs. The area occupied by Kernza in the USA in 2014 was approximately 87 hectares and doubled to 170 hectares in 2016. For further growth of the areas occupied under this crop, information on optimal establishment practices, assessment of forage nutritive value, ways to maintain grain yields over years, and weed management is needed (Lanker et al., 2020).

## The usage of IWG grain for increasing the nutritional and biological value of bread and baked goods

An important aspect of the popularization of IWG in America and Europe was the use of Kernza grain for food production (Zhang X. et al., 2017). Bakery products, crackers, cereals, snacks produced on the basis of wheatgrass grain have a sweet nutty taste. The companies General Mills and Patagonia Provisions produce the wheatgrass grain goods under the trademark Kernza®, which belongs to the Land Institute. Currently, these companies are expanding the markets for these products. A chain of Birch Wood cafes has been opened in Minneapolis, serving tortillas and pancakes baked from flour of wheatgrass Kernza (Springmann et al., 2018).

Studies have been conducted for evaluation of technological characteristics of wheatgrass grain. The results have been used for the development of food products. The IWG grain quality is not inferior to wheat grain, but at the same time, there are significant differences (Becker et al., 1991).

IWG is characterized by a high protein and fiber content in whole grain flour – 20 and 16.4 %, while in whole grain wheat flour their content is 13 and 11 %, respectively (Rahardjo et al., 2018). Protein has more essential amino acids compared to wheat, in particular, 1.4 times more cysteine and methionine (Becker et al., 1991). The results of a 3-year research on the IWG variety Sova (Th. intermediate) under conditions of the southern forest-steppe of Western Siberia showed that the protein content in grain varied from 18.5 to 20.5 %. For the third year of the variety’s production, the protein content increased by 2 %. This is probably related to an increase in the total number of important agronomic groups of microorganisms in the rhizosphere under the variety Sova, development of more powerful root system, and weather conditions (Shamanin et al., 2021).

Wheatgrass glutenin proteins contain fewer high-molecularweight glutenin subunits (HMW-GS), which are similar in structure to wheat HMW-GS (67–120 kDa), but have a lower weight – 45–90 kDa (Zhang Х. et al., 2014). The deficiency in HMW-GS with a molecular weight of >60 kDa in wheatgrass grain causes a weak gas-holding capacity and dough elasticity, which, in turn, leads to low bread making quality (Marti et al., 2016).

Due to the small size of wheatgrass seeds, they contained significantly less starch (46.7 %) compared to wheat (72 %), as well as more albumin and globulin proteins in the aleurone layer. However, during domestication, the seed weight was increased by 23 % (DeHaan et al., 2018), which led to an increase in the endosperm proportion in the seed and, accordingly, starch. The technological and digestive properties of starch depend on its content. The management of its components, amylose and amylopectin can be regulated using combinations of alleles of Wx genes in Th. intermedium (Klimushina et al., 2020). In contrary to wheat starch, wheatgrass starch has a higher proportion of long amylose chains, a lower gelatinization temperature, which reduces the starch viscosity and retrograde and makes it suitable for the production of baked goods with a lower glycemic index (Zhong et al., 2019). Th. intermedium grain can also be used in a mixture with hard red wheat grain for the production of baked goods with a low glutenin content (Marti et al., 2015; Rahardjo et al., 2018).

Mixing wheatgrass grain flour and durum wheat grain flour in a ratio of 50:50 contributes to a good balance between the functional characteristics and digestive properties of baked goods. Particularly, cookies made of wheatgrass grain flour had the same quality as cookies made of ordinary wheat flour. In addition, the increased content of dietary fibers and antioxidants in wheatgrass flour baked goods makes them especially useful for human health (Marti et al., 2016).

## IWG variety Sova as alternative to perennial wheat

**Fig. 1.**
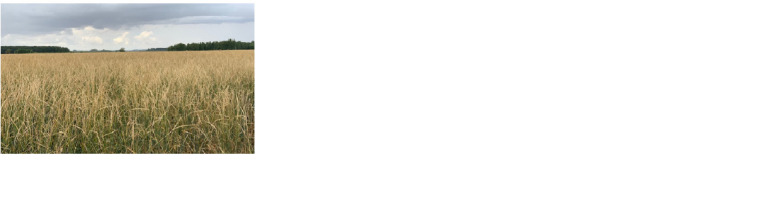
Variety Sova of the 2nd year reproduction in JSC ”Niva” of Pavlograd region, Omsk oblast, 2020.

**Fig. 2.**
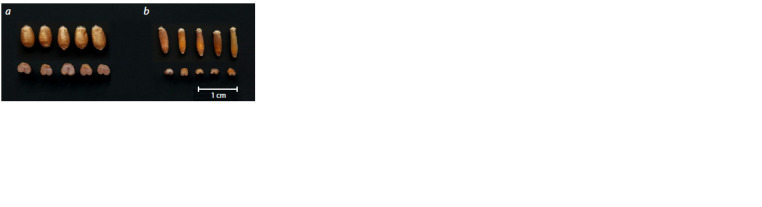
Grain of spring bread wheat Triticum aestivum L. variety Pamyati Azieva (a) and grain of wheatgrass Thinopyrum intermedium (Host) Barkworth & D.R. Dewey variety Sova (b), experimental field of Omsk SAU.

Omsk State Agrarian University initiated a study on the cultivation of perennial wheat samples obtained from the international CIMMYT collection and wheatgrass populations developed at the Land Institute. The city of Omsk became one of the sites among multilocation experiments of perennial crops germplasm, the results of which are presented in the article of R.C. Hayes et al. (2018). The variety Sova was developed by mass selection of overwintered biotypes from the Th. intermedium population received from the Land Institute. Several selection rounds were carried out on the basis of traits of winter hardiness and spike productivity. The productivity components of 100 spikes were evaluated according to the following characteristics: spike weight and length, the number of spikelets and grains per spike, the number of grains per spikelet, grain weight per spike. A synthetic population adapted to the conditions of the southern forest-steppe of Western Siberia was formed by directed pollination of the selected biotypes. In 2020, the large-grain wheatgrass variety Sova was included in the State Register of Breeding Achievements Allowed for Use and recommended for cultivation in all regions of Russia (Fig. 1, 2).

The variety Sova can be cultivated as a dual-use crop – for grain and forage. The average grain yield was 0.92 t/ha, green mass – 21.0 t/ha, and hay – 7.1 t/ha (Shamanin et al., 2021). Omsk State Agrarian University produces original seeds of the variety Sova with subsequent reproduction of the elite category seeds in three basic farms of Omsk State Agrarian University: “Triticum”, “Niva”, and “Govin”. In 2020, about 5 seed tons of the variety Sova were produced for farmers in the Omsk region. The average grain yield in the southern forest-steppe and steppe zones of the Omsk region was 0.4–0.6 t/ha.

Despite some progress made in the implementation of individual breeding programs, there are many tasks that require further selection solutions to improve the efficiency of wheatgrass cultivation as a perennial grain crop. First of all, it is necessary to increase the yield of wheatgrass grain. The grain yield of wheatgrass is lower than that of spring wheat, because part of its energy is spent on the development of the root system and branching after overwintering. A further increase in the wheatgrass grain yield can be achieved by repeated selections of forms with a smaller plant length and a smaller number of grains per spike, which seems advisable to increase the thousand kernel weight (TKW). This is evidenced by the research results, in which a negative correlation between the TKW and the plant height (r = –0.3, p = 0.05), between the TKW and the number of grains per spike (r = –0.5, p = 0.01) was noticed (Shamanin et al., 2021). The usage of genomic technologies and molecular mapping for the selection of genotypes with valuable traits will greatly contribute to improving the efficiency of breeding for increasing the grain yield of this perennial crop.

Efficient seed production technologies and agrotechnical methods of wheatgrass cultivation in specific agro-climatic zones are also a reserve for increasing the yield of this crop. For producing bread and bakery goods made of wheatgrass grain with functional properties, it is necessary to develop technologies for the food industry and market the demand for this product by the population, which will allow to form a stable demand for this crop on the market.

## Conclusion

The above review of world research shows that IWG is a culture with great production potential, beneficial ecological properties and valuable grain for functional food. Cultivation of Th. intermedium and other perennial crops – sorghum, rice, barley, Silfium, meadow and pasture grasses in agriculture will provide not only ecological, but also social and economic benefits. This is also important due to challenges associated with the climate warming, the necessity to reduce the greenhouse effect, in agricultural production as well. The grain of IWG can be used for bakery and confectionery products with improved nutritional value, and the whole plant can be used for biomass, hay, and haylage. IWG has increased resistance to many negative biotic and abiotic environmental factors, forms a strong root system that improves plant water consumption, reduces nutrient losses in the soil and carbon emissions. The wheatgrass varieties Kernza and Sova developed at the Land Institute (USA) and at Omsk State Agrarian University (Russia) indicate good prospects for breeding improvement of this crop. Considering that the variety Sova is significantly inferior to cultivated annual cereals in grain yield, new breeding programs aimed to increase the thousand kernel weight and the manufacturability of cultivation in specific agro-climatic zones are needed. Active marketing and development of technologies for production of the wheatgrass grain for functional food production are necessary to popularize a new crop on the market.

## Conflict of interest

The authors declare no conflict of interest.
